# Global Research Trends in Treatments for Ureteropelvic Junction Obstruction: A Multi-Database Bibliometric Analysis of the Past Two Decades

**DOI:** 10.3390/healthcare14203533

**Published:** 2026-10-20

**Authors:** Lei Xiang, Qiyu He, Lede Lin, Liang Zhou

**Affiliations:** Department of Urology, Institute of Urology (Laboratory of Reconstructive Urology), West China Hospital, Sichuan University, Chengdu 610041, China

**Keywords:** ureteropelvic junction obstruction, treatment, bibliometric analysis, surgical plan

## Abstract

**Background:** Ureteropelvic junction obstruction (UPJO) is one of the most common causes of hydronephrosis in children. Over the last several years, treatment methods have evolved toward less invasive procedures. However, bibliometric analysis of UPJO treatment remains limited. We applied a multi-database bibliometric approach to describe the current status and recent and emerging research trends in UPJO management. **Methods:** We retrieved studies on the treatment of UPJO from PubMed, Scopus, Embase, Web of Science, and Cochrane Library, spanning from 1 January 2006 to 31 December 2025; all five databases were searched on 20 January 2026. Records were merged in EndNote X9 (Clarivate Analytics, Philadelphia, PA, USA), deduplicated using Author, Year, and Title, and manually verified using DOI and minor title/author variants. Web of Science was used as the single source for citation counts and cited-reference data in citation-based analyses. Of the 849 included publications, 736 were matched to Web of Science, and 728 of these contributed cited-reference data to the co-citation analysis. We analyzed data using CiteSpace (v6.2.R.3; one-year slices; g-index k = 25) together with the bibliometrix R package (v4.6). **Results:** A total of 849 publications, including 761 original articles and 88 reviews, from 55 countries were included in the analysis, with the United States and China leading in publication volume. Annual citation activity generally increased with year-to-year variation. A total of 3545 authors participated in these publications, among whom Lorenzo AJ, Noh PH, and Peters CA had the highest total citation counts. Wroclaw Medical University, Peking University and University of Toronto had the highest publication counts among institutions. The funding analysis indicated that the National Natural Science Foundation of China, United States Department of Health and Human Services, and National Institutes of Health were the top three funding bodies by publication count. **Conclusions:** Management-related research on UPJO is active, with current emphasis on technological innovation, full-cycle management, and complex case management. Robot-assisted pyeloplasty remains a prominent bibliometric theme in the recent literature.

## 1. Introduction

Ureteropelvic junction obstruction (UPJO) occurs when the area that connects the renal pelvis to the ureter becomes blocked, and it is one of the most common causes of hydronephrosis in children, with an incidence of 1 in 2000 live births [1,2,3]. Hydronephrosis in children has heterogeneous causes, including congenital anomalies of the kidney and urinary tract, upper urinary tract obstruction at the ureteropelvic or ureterovesical junction, vesicoureteral reflux, lower urinary tract obstruction such as posterior urethral valves, and transient or non-obstructive dilatation [2,3]. Untreated UPJO, whether congenital or acquired, can lead to significant renal dysfunction and, in severe cases, loss of function in the affected kidney [4]. The past few decades have witnessed increasing use of minimally invasive surgical techniques [5,6]. Open pyeloplasty remains an established therapy, while laparoscopic and robotic-assisted approaches have been introduced with favorable perioperative and cosmetic outcomes in selected patients [7,8,9].

Bibliometrics is an important tool for analyzing publication productivity and new trends in science and can provide quantitative and qualitative information on authors, keywords, journals, nations, institutions, and citations [10,11,12,13]. A recent bibliometric study by Qiu et al. focused specifically on robot-assisted pyeloplasty using Web of Science [14], whereas the broader UPJO treatment literature across conservative, open, endoscopic, laparoscopic, and robotic approaches has not been characterized using multiple databases. We therefore aimed to characterize publication and citation activity, identify the main contributors using explicit bibliometric metrics, map collaboration and co-citation structure, and describe thematic evolution and recent research trends. The study was designed to identify research patterns rather than predict clinical practice or comparative treatment effectiveness.

## 2. Materials and Methods

### 2.1. Data Sources and Retrieval Strategies

**Figure 1:**
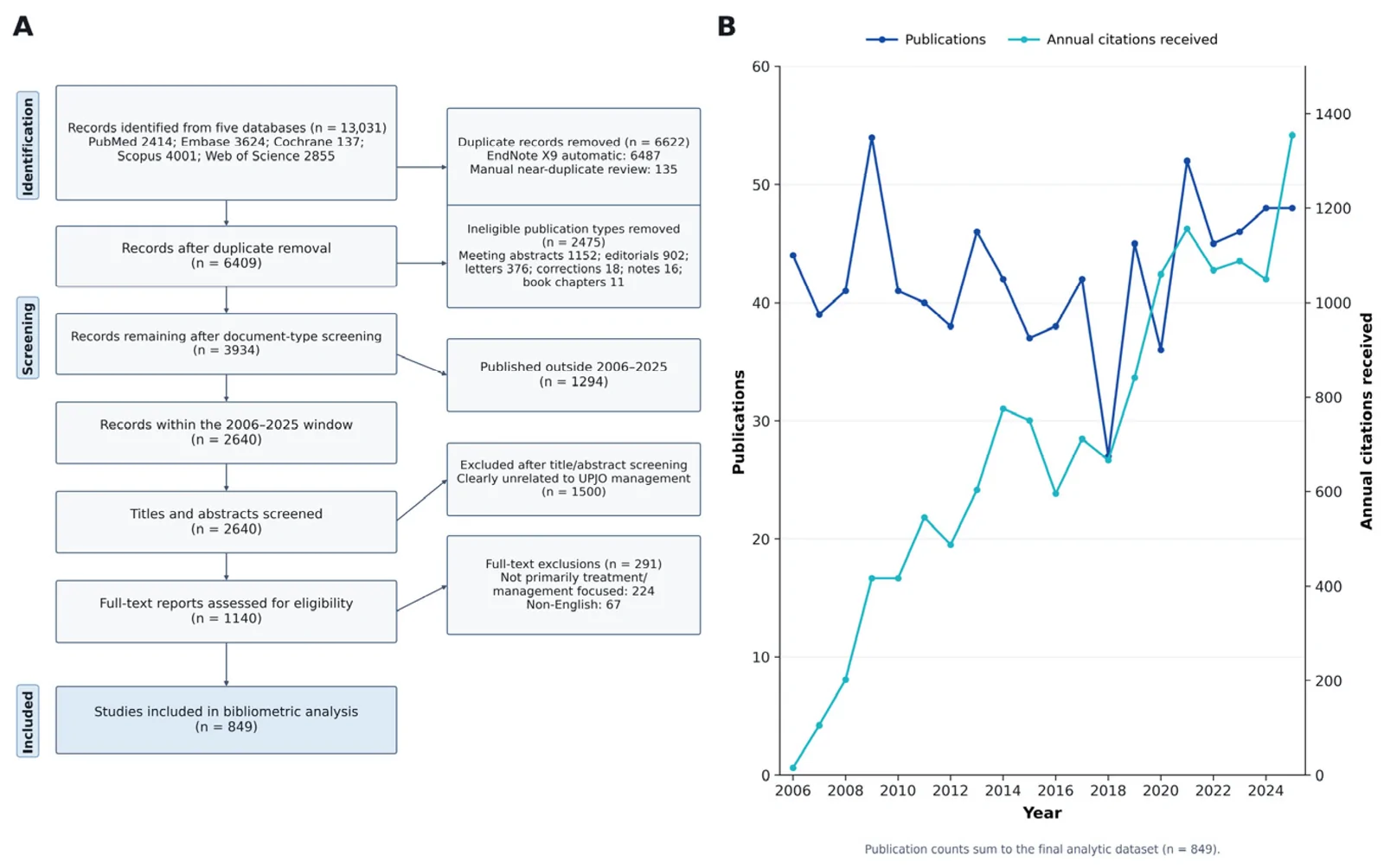
Literature selection process and annual publication/citation activity in ureteropelvic junction obstruction (UPJO) treatment research, 2006–2025. (**A**) PRISMA-style flow diagram of record identification, deduplication, screening, eligibility assessment, and inclusion. (**B**) Annual publication output and annual citations received by the included corpus.

We comprehensively collected literature on the treatment of UPJO from PubMed, Scopus, Embase, Web of Science, and Cochrane Library, covering 1 January 2006 to 31 December 2025. All five databases were searched on 20 January 2026. The search strings were executed without language or document-type restrictions; the publication-year window was applied after export, and English-language/article-or-review eligibility was applied during screening. Records were exported as PubMed NBIB, Embase RIS, Scopus CSV, Web of Science plain text (Full Record and Cited References), and Cochrane RIS files. Two authors independently carried out the searches on the same day employing terms related to UPJO treatment. Disagreements were resolved after discussion with a third reviewer. The literature screening process is shown in Figure 1A, and detailed search strategies and results are shown in Supplementary Table S1, with PRISMA-S providing guidance on search reporting [15]. References published in 2026 were used only for contextual discussion and were not included in the bibliometric dataset.

Eligibility for inclusion was defined by the following criteria: (1) studies focusing on the treatment or management of UPJO; (2) document types of original research articles or review articles; and (3) English language. Exclusion criteria were: (1) studies not primarily related to UPJO treatment, such as etiology or diagnosis without a therapeutic focus; (2) non-English publications; and (3) meeting abstracts, editorial materials, letters, corrections, notes, book chapters, and non-peer-reviewed materials. After duplicate, document-type, and publication-year exclusions, two reviewers independently screened 2640 titles and abstracts; 1500 records clearly unrelated to UPJO management were excluded. The remaining 1140 reports underwent full-text assessment, and 291 were excluded because treatment/management was not the primary focus (*n* = 224) or the report was not in English (*n* = 67), leaving 849 publications. Formal inter-reviewer kappa was not calculated; disagreements were resolved by consensus with a third reviewer.

After exporting records from all five databases, duplicates were identified in EndNote X9 using Author, Year, and Title [16]. DOI was used during manual verification, and records lacking a DOI or showing minor title or author variations were compared manually by two reviewers. The five database searches identified 13,031 records in total. First-pass EndNote X9 duplicate detection removed 6487 records; manual review of records with missing DOI or minor title/author variations removed a further 135, leaving 6409 unique records. Disagreements were resolved by consensus with a third reviewer. Eventually, 849 publications, including 761 original articles and 88 reviews, were included in the bibliometric analysis.

### 2.2. Data Acquisition and Analysis

The 849 selected publications were consolidated into a single harmonized dataset containing bibliographic information on publication and citation counts, countries, institutions, authors, journals, co-cited references, funding agencies, and keywords. Author names were standardized to surname plus initials, institution names were mapped to a controlled parent-institution form, journal names were standardized by ISSN/full title, and country names were harmonized; missing affiliation, funding, keyword, and cited-reference fields were not imputed. Web of Science Core Collection was used as the single source for article-level citation counts and cited-reference data used in citation-ranked and co-citation analyses, and citation values from different databases were not summed or averaged. Linkage to Web of Science was attempted first by DOI; records without an exact DOI match were matched by title and checked against publication year and author information. Of the 849 included publications, 736 (86.7%) were matched and used for citation-based analyses. Among these, 728 contained cited-reference data and were used for co-citation analysis. Unmatched records remained in analyses that did not require citation data, and citation information from other databases was not substituted for missing Web of Science data. We generated visual atlases using CiteSpace (v6.2.R.3) for author, institution, country, co-citation, keyword co-occurrence, clustering, timeline, and burst analyses [17]. All analyses used the 2006–2025 time span with a slice length of 1 year, g-index selection (k = 25), LRF = 3.0, L/N = 10, LBY = 5, and e = 1.0. Term sources for keyword analyses were Title, Abstract, Author Keywords, and Keywords Plus/database-supplied keywords. Pruning was not applied to author, institution, country, or co-citation networks; Pathfinder pruning was applied to the keyword network. Cluster labels were generated using the log-likelihood ratio method, and burst detection used the Kleinberg algorithm (γ = 1.0; minimum duration = 2 years). bibliometrix (v4.6) [18] was used for descriptive summaries and metadata extraction (convert2df, biblioAnalysis, summary, and metaTagExtraction). Annual trend and geographic visualizations were generated in R (version 4.2.1) using ggplot2/tidyverse and, for the world map, sf, rnaturalearth, countrycode, and scico. Journal impact factors and quartiles were obtained from the 2024 Journal Citation Reports. CiteSpace network figures were exported using the software-generated display labels.

## 3. Results

### 3.1. Annual Publication and Citation Trend Analysis

The number of publications per year and the frequency of literature citations are important indicators of research activity. Ultimately, 849 studies were included, consisting of 761 original articles and 88 review articles. Citation analyses included 736 Web of Science-matched publications; 728 of these contributed cited-reference data to the co-citation analysis. Annual publication output fluctuated over the past two decades, while annual citations received by the included corpus generally increased with year-to-year variation (Figure 1B). The citation series represents citations received during each calendar year rather than cumulative citations grouped by publication year.

### 3.2. Country and Region

**Figure 2:**
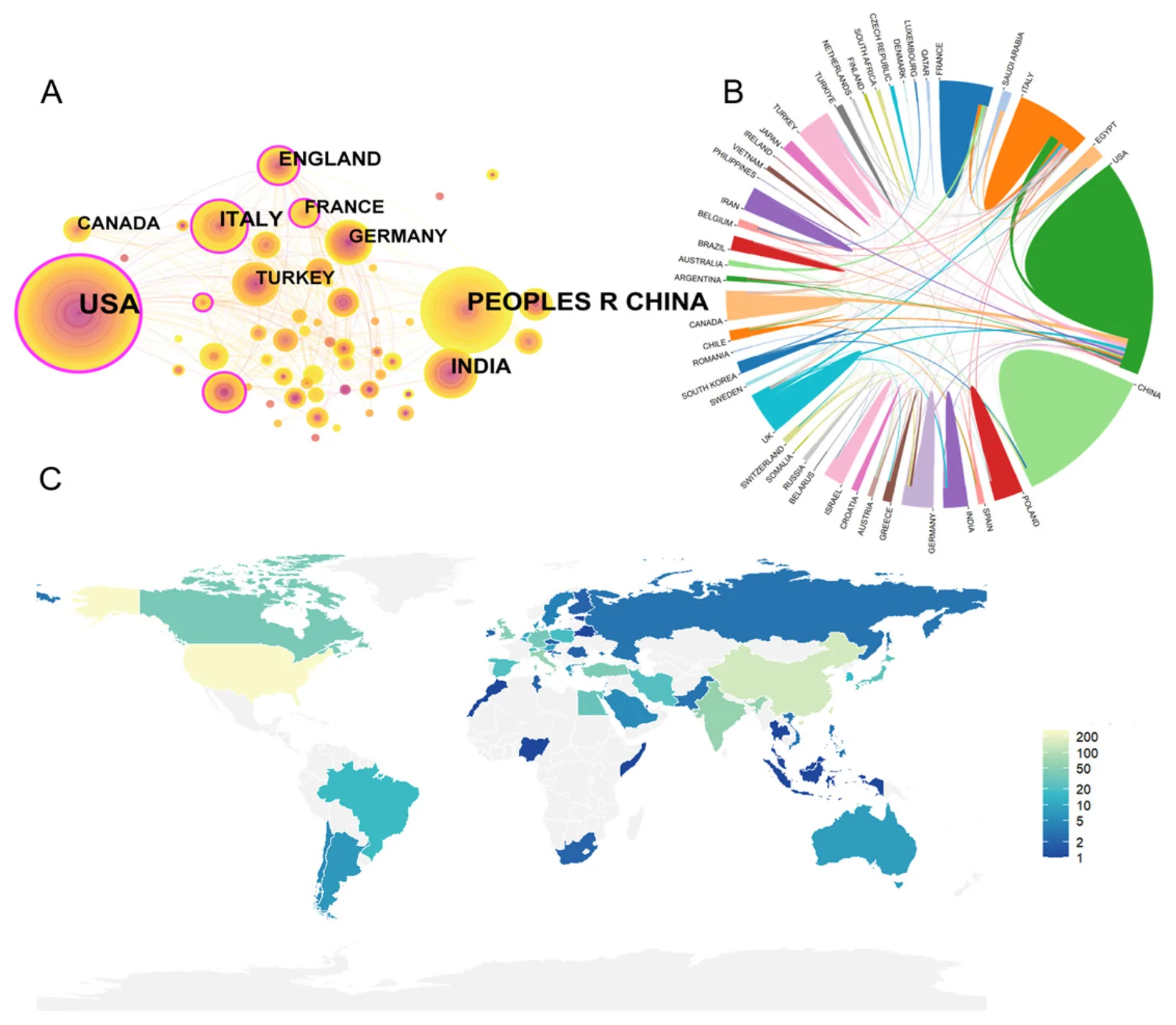
National collaboration and distribution in the field of ureteropelvic junction obstruction (UPJO) treatment (2006–2025). (**A**) Co-occurrence network of major countries involved in UPJO treatment research. (**B**) Sankey diagram illustrating publication output distribution and international collaboration in UPJO treatment research. (**C**) Global distribution of publication output by country in the field of UPJO treatment.

Among all included studies in the field of UPJO treatment, the United States and China had the largest publication outputs and occupied prominent positions in the global collaboration network (Figure 2A). Countries such as the United Kingdom, Germany, Italy, Turkey, and India also serve as important research nodes. The United States has intensive collaborative links with multiple countries, including China, Turkey, Germany, and the United Kingdom, showing broad international collaboration. China’s most prominent collaborative link is with the United States, while it also maintains stable academic collaborations with other countries (Figure 2B).

The literature in the treatment of UPJO in urological diseases is geographically dispersed across 55 countries and regions (Figure 2C). In this field, North America (the United States and Canada), Asia (China, India, Japan, and South Korea), and Europe (the United Kingdom, Germany, Italy, and Turkey) had relatively high research output. In contrast, most regions in Africa, South America, and Oceania have relatively low research output, indicating uneven global representation in the indexed literature.

### 3.3. Author and Institution

**Figure 3:**
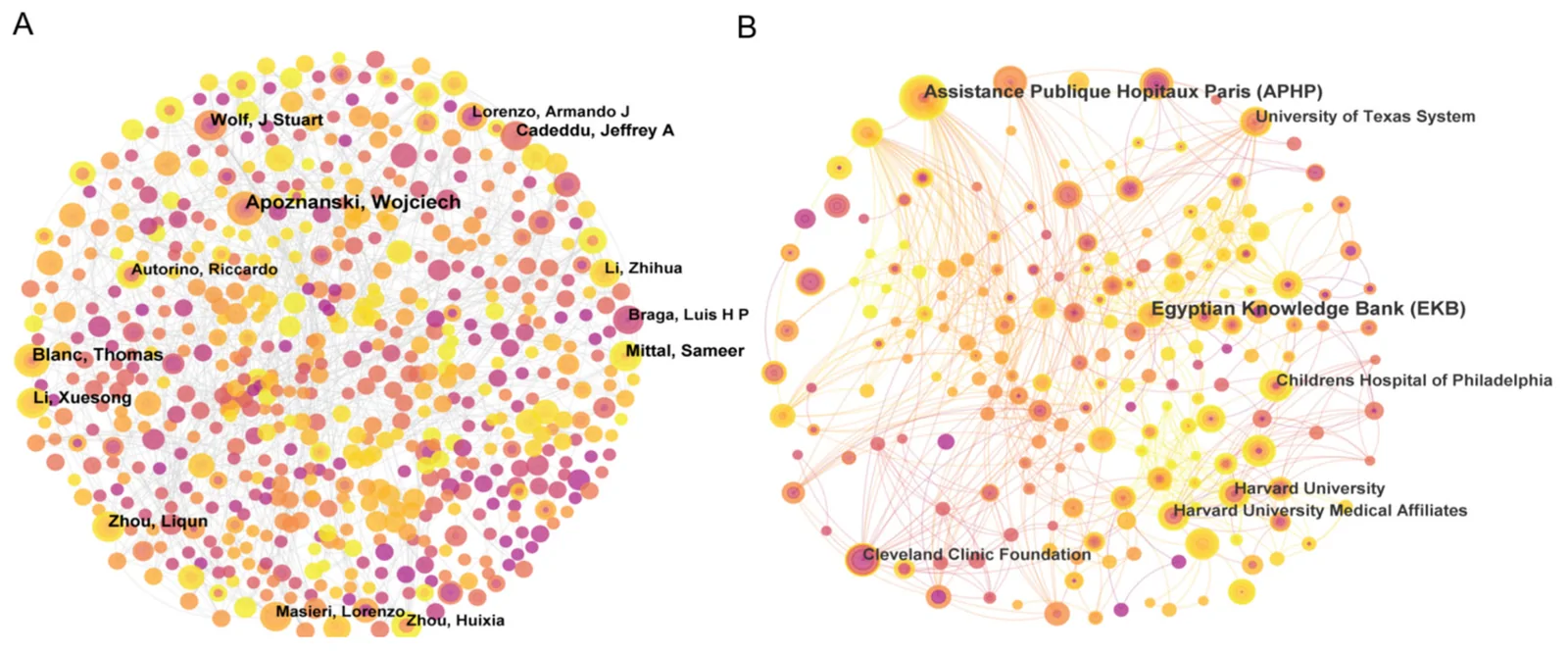
Author and institution collaboration networks in the field of ureteropelvic junction obstruction (UPJO) treatment (2006–2025). (**A**) Author collaboration network in UPJO treatment research. (**B**) Institution collaboration network in UPJO treatment research.

The author and institutional collaboration networks reveal a multi-national research network with several relatively stable collaboration groups and cross-group connections (Figure 3A). Supplementary Table S2 ranks authors by total citation count: Lorenzo AJ, Noh PH, and Peters CA had the highest citation counts. Highly productive authors in the collaboration network included Apozhanski, Wojciech, Wolf, J Stuart, Lorenzo, Armando J, Cadeddu, Jeffrey A, and Autorino, Riccardo. Chinese scholars such as Li, Zhihua, Li, Xuesong, Zhou, Liqun, and Zhou, Huixia were also prominent in the network.

Institutions with the highest publication counts included Wroclaw Medical University, Peking University, and the University of Toronto (Supplementary Table S3). The Egyptian Knowledge Bank (EKB) appears as a visible node in the collaboration network (Figure 3B). The distributed connections indicate international institutional collaboration.

### 3.4. Funding Agency

The analysis of funding agencies showed that UPJO management research was supported by a diverse range of organizations (Supplementary Table S4). The National Natural Science Foundation of China had the largest publication count, followed by the United States Department of Health and Human Services and the National Institutes of Health. These counts describe how frequently funding bodies were reported in the included publications and do not quantify comparative funding magnitude or effectiveness.

### 3.5. Core Journal

The ten journals with the largest publication counts are listed in Supplementary Table S5 and contributed 60.4% of the publications in this area. Journal of Endourology had the highest publication count. Although Journal of Urology ranked fourth by publication count (63 articles), it had the highest total citations (1345) within this top-ten set. The 2024 Journal Citation Reports impact factors and quartiles are listed in Supplementary Table S5.

### 3.6. Co-Citation and Citation

**Figure 4:**
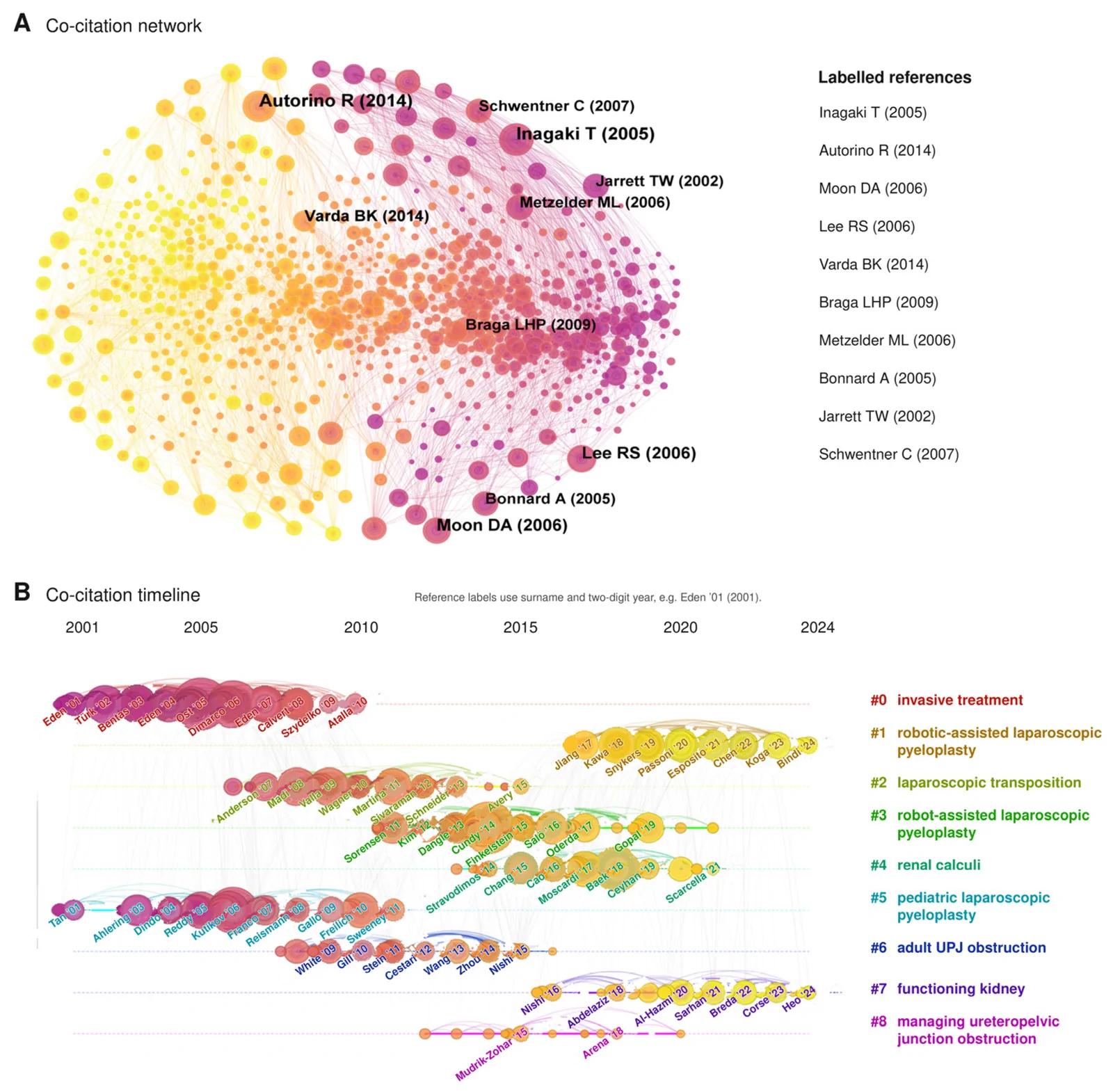
Co-citation network and timeline analysis of research on ureteropelvic junction obstruction (UPJO) treatment. (**A**) Co-citation network with selected references labelled by first author and publication year [20,21,22,23,24,25,26,27,28,29]. (**B**) Timeline view of the co-citation clusters. Reference labels in (**panel B**) show the first author’s surname and two-digit publication year.

Two studies are considered to have a co-citation relationship when they are simultaneously cited in one or more later papers [12,19]. Using CiteSpace, we constructed a literature co-citation network map (Figure 4A) comprising 787 nodes, 4002 links, and a density of 0.0129; the largest connected component contained 744 nodes (94.5%), and no pruning was applied. The timeline visualization had modularity Q = 0.6578 and weighted mean silhouette S = 0.8736. It showed earlier clusters focused on invasive treatment and pediatric laparoscopic pyeloplasty and later clusters including robotic-assisted laparoscopic pyeloplasty and functioning kidney (Figure 4B). The top ten highly co-cited publications and the top ten publications by total citation count are presented separately in Table 1 and Table 2.

### 3.7. Keyword Clustering and Bursting Detection

**Figure 5:**
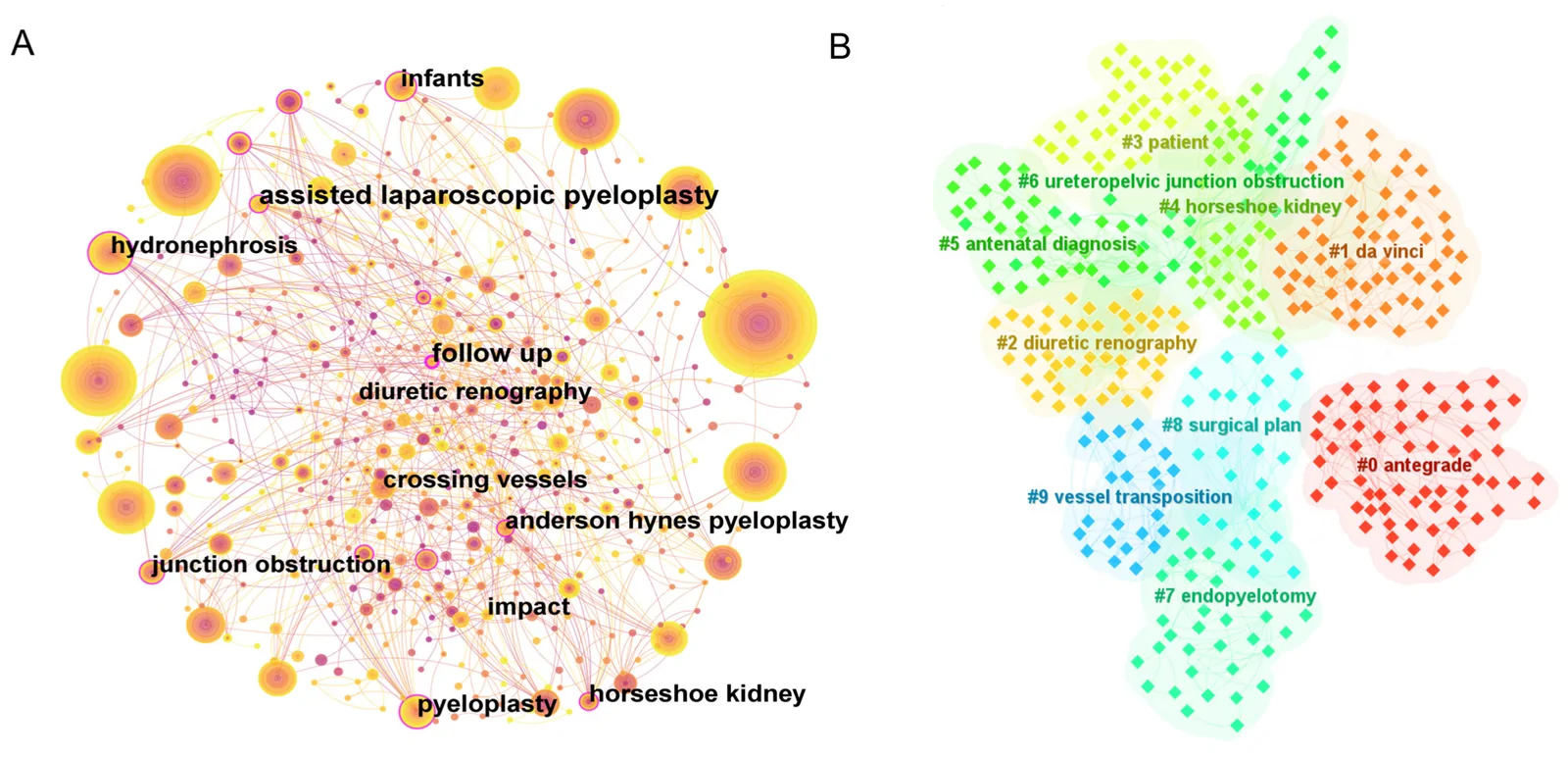
Keyword co-occurrence and topic clustering in the field of ureteropelvic junction obstruction (UPJO) treatment (2006–2025). (**A**) Keyword co-occurrence network in UPJO treatment research. (**B**) Topic clustering of keywords in UPJO treatment research.

The keyword co-occurrence network in UPJO treatment research is shown (Figure 5A), and keyword clustering analysis revealed ten major clusters in the field of UPJO treatment research, including #0 12 year, #1 da vinci, #2 diuretic renography, #3 patient, #4 horseshoe kidney, #5 antenatal diagnosis, #6 ureteropelvic junction obstruction, #7 endopyelotomy, #8 surgical plan, #9 vessel transposition (Figure 5B). The keyword network comprised 518 nodes and 1037 links (density 0.0077) with Pathfinder pruning. The modularity (Q) value was 0.7349, and the weighted mean silhouette (S) value was 0.906. These clusters reflect the research hotspots and structure of the UPJO treatment field, demonstrating that technological innovation, full-cycle management, and complex case management are the three major research directions in the current field. Technological innovation is primarily represented by #1 da Vinci, which centers on robotic-assisted surgical techniques, and #7 endopyelotomy, focusing on endoscopic and minimally invasive incision methods. These clusters describe a major body of literature on minimally invasive and reconstructive techniques. Full-cycle management encompasses clusters that cover the entire disease course from prenatal detection to long-term follow-up. #5 antenatal diagnosis highlights research on early detection and fetal management, while #2 diuretic renography relates to functional imaging for diagnosis and postoperative assessment. #0 “12 year” and #6 ureteropelvic junction obstruction represent additional thematic groupings identified by CiteSpace. Complex case management involves anatomically or clinically challenging scenarios. #4 horseshoe kidney and #9 vessel transposition address congenital anomalies and aberrant vessels, often requiring tailored surgical strategies. #8 surgical plan emphasizes individualized preoperative planning, while #3 patient reflects patient-centered considerations such as risk stratification and quality of life.

**Figure 6:**
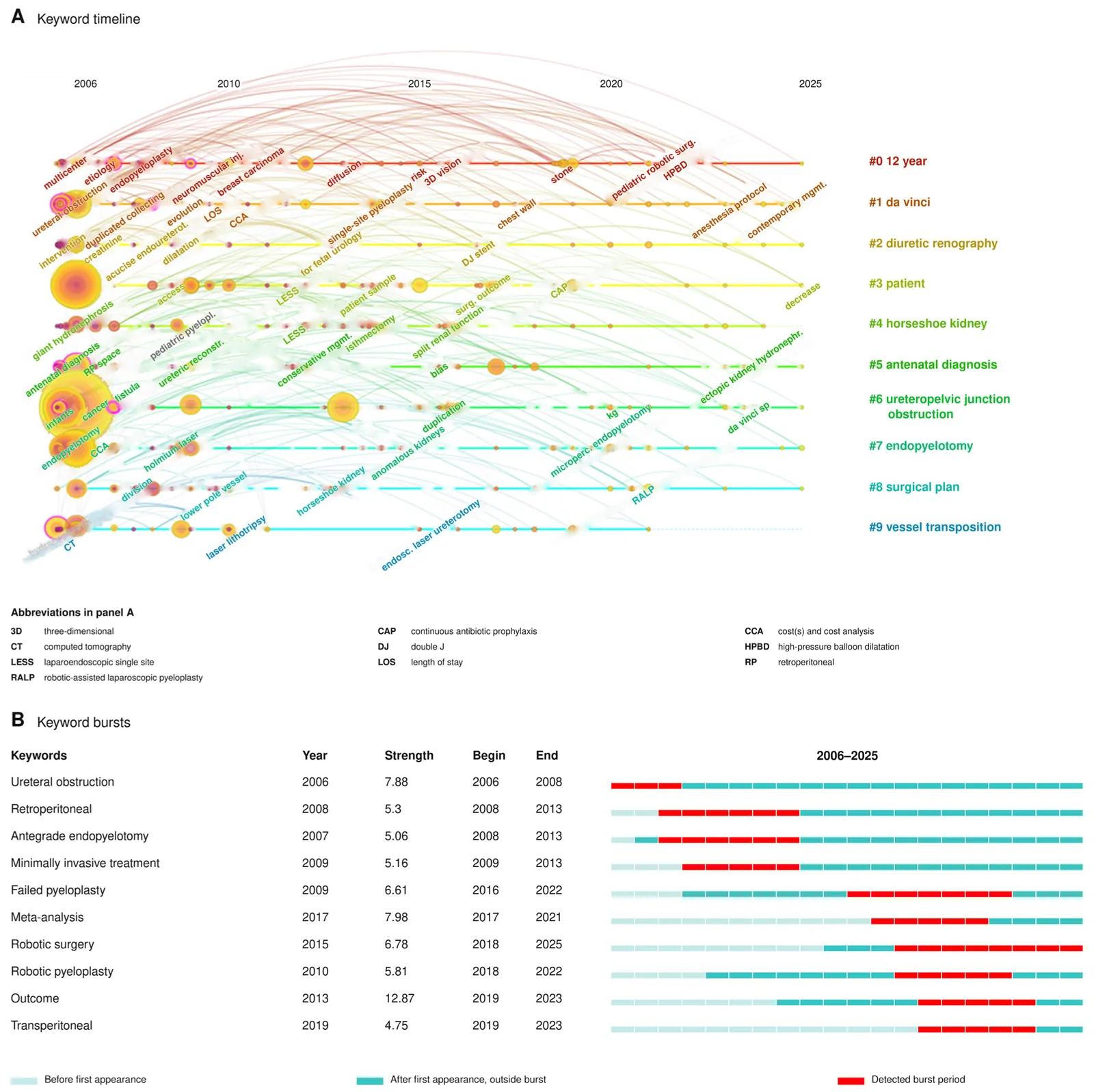
Keyword timeline and burst analysis of research on ureteropelvic junction obstruction (UPJO) treatment (2006–2025). (**A**) Timeline view of the keyword clusters. Some keyword labels are abbreviated for readability; the abbreviations are defined in the figure. (**B**) Ten keywords with the strongest bursts in occurrence frequency. Light cyan indicates years before a keyword first appeared in the dataset, dark cyan indicates subsequent years outside the detected burst period, and red indicates the detected burst period.

Time-based keyword evolution analysis in UPJO treatment research is shown (Figure 6A), and burst detection analysis identified the top ten keywords with the highest citation burst intensity from 2006 to 2025 (Figure 6B). Among them, “outcome” had the highest burst intensity (12.87), showing concentrated research attention to treatment outcomes. “Robotic surgery” and “Robotic pyeloplasty” had the longest burst periods, indicating sustained research attention to robotic approaches. Other keywords with significant burst intensity included “Meta-analysis” (7.98) and “ureteral obstruction” (7.88), reflecting attention to evidence synthesis and disease evaluation. The recent emergence of “failed pyeloplasty” and “Robotic pyeloplasty” highlights recent attention to salvage management and robotic pyeloplasty. Artificial intelligence, surgical training curricula, and cost-effectiveness were not among the reported top burst keywords.

## 4. Discussion

### 4.1. Current Global Research Landscape of UPJO Treatment

This study systematically mapped the research status of UPJO treatment over the past two decades (2006–2025), covering 849 publications from 55 countries. The United States and China had the largest publication outputs and occupied prominent positions in the collaboration network. Lorenzo AJ, Noh PH, and Peters CA had the highest total citation counts among authors. Wroclaw Medical University, Peking University, and the University of Toronto had the highest publication counts among institutions. The concentration of work in a limited number of countries and institutions, while many regions have lower representation, indicates geographic imbalance in the indexed literature.

The funding agency analysis shows that the National Natural Science Foundation of China, the U.S. Department of Health and Human Services, and the National Institutes of Health were the three most frequently reported funding bodies. Journal of Urology, despite ranking fourth in publication volume, had the highest total citations within the ten most productive journals, while Journal of Endourology led in publication volume.

Our findings align with a recently published bibliometric analysis by Qiu et al. [14], which focused specifically on robot-assisted pyeloplasty (RAP) for UPJO from 2001 to 2025. In that study, the United States and China also emerged as the most productive countries, with thematic evolution shifting from feasibility and perioperative outcomes toward redo procedures and complex anatomies. However, our study differs in scope: we included all treatment modalities (open, laparoscopic, robotic, endoscopic) rather than limiting to robotic procedures. Our search strategy incorporated five databases, whereas Qiu et al. relied exclusively on Web of Science. This may explain why our analysis identified a more diverse set of core authors and institutions, including contributions from Poland, Turkey, and India. Both studies identify robotic pyeloplasty as a prominent research topic.

### 4.2. Evolution of Research Hotspots and Technological Progress

Many studies have focused on UPJO management because of its importance in pediatric urology [2,36,37,38,39]. Earlier studies addressed coexisting vesicoureteral reflux and UPJO [40], percutaneous pyelotomy [41], long-term follow-up after pyeloplasty [42], and the role of open pyeloplasty [43]. The clinical literature has progressed from open reconstruction and early endoscopic approaches to laparoscopic and robot-assisted techniques [38,39,44,45,46,47,48,49,50,51,52,53,54,55]. Studies of minimally invasive approaches have reported perioperative and cosmetic advantages in selected patients, while systematic reviews and comparative studies continue to evaluate relative outcomes. Table 3 summarizes the cited clinical literature to provide context for the bibliometric findings.

The co-citation network and keyword clustering analysis show the temporal development of UPJO treatment research. The field has evolved from a foundational stage focusing on invasive treatment and pediatric laparoscopic pyeloplasty to a current stage centered on robotic-assisted laparoscopic pyeloplasty and functional kidney protection. This pattern reflects a shift in research emphasis over time.

Keyword burst detection identifies topics with concentrated recent attention. “Outcome” has the highest burst intensity, showing strong research attention to treatment outcomes. The long burst period of “robotic surgery” and “robotic pyeloplasty” indicates sustained research attention to robotic approaches. The emerging keyword “failed pyeloplasty” highlights recent attention to salvage management.

The three major research clusters identified constitute the current research framework of UPJO treatment: technological innovation, full-cycle management, and complex case management. Technological innovation is embodied in the continuous optimization of minimally invasive surgical techniques. Full-cycle management covers the disease process from antenatal diagnosis to long-term postoperative follow-up. Complex case management focuses on anatomically atypical patients such as those with horseshoe kidney and vessel transposition.

### 4.3. Interpretation of Bibliometric Findings and Clinical Context

Robot-assisted pyeloplasty is highly visible in the recent UPJO literature. Clinical comparisons and systematic reviews cited in this manuscript report outcomes for open, laparoscopic, and robotic approaches; Table 3 summarizes this literature as clinical context alongside the bibliometric findings.

Clusters related to antenatal diagnosis, diuretic renography, outcome, and patient-centered themes show that the literature spans diagnosis, intervention, and follow-up across the disease course.

The geographic network shows broad international participation, although publication output remains concentrated in a limited number of countries and institutions.

Cost-effectiveness, surgical training curricula, and artificial intelligence were not among the leading burst terms identified in this analysis and are discussed as potential topics for future clinical and implementation research.

### 4.4. Limitations

Several limitations should be acknowledged. First, the exclusion of non-English literature may omit relevant research from some regions. Second, citation counts are influenced by publication age, journal visibility, language, database coverage, and self-citation, and high citation counts do not necessarily indicate high clinical value. We used Web of Science as the single source for citation counts and cited-reference data, which avoids cross-database summation but can underrepresent records not indexed there. Citation analyses therefore included 736 of the 849 publications, and 728 contributed cited-reference data to the co-citation analysis. Third, author, institution, journal, and country names vary across databases, and residual entity-splitting or merging may remain despite harmonization. Fourth, the reconciled workflow removed 6487 records during first-pass EndNote X9 duplicate detection and a further 135 near-duplicates during manual verification; manual verification was based on DOI, title, author, year, and journal fields when available. Fifth, the analysis period ended on 31 December 2025; references published in 2026 were used only for contextual discussion. Finally, bibliometric analyses identify patterns of research attention and intellectual structure but cannot determine comparative treatment effectiveness, preferred management, or future developments. Detailed database-integration rules and network settings are summarized in Supplementary Table S6.

### 4.5. Future Research Directions

The bibliometric findings directly support continued study of outcomes, robotic pyeloplasty, failed pyeloplasty, complex anatomy, and longitudinal assessment. Beyond these observed themes, future clinical research could examine the following questions. Cost-effectiveness analysis of robotic-assisted laparoscopic pyeloplasty could clarify value across different health-care settings. Standardization of surgical training curricula could support competency assessment. Integration of artificial intelligence in UPJO treatment could be evaluated for imaging, preoperative planning, intraoperative navigation, and outcome prediction. Research on complex cases such as failed pyeloplasty and congenital malformations requires further investigation. These topics are proposed research directions based on clinical relevance and technological development; they were not identified among the top keyword bursts in this analysis.

## 5. Conclusions

This bibliometric analysis describes the global research landscape, hotspots, and evolution trends of UPJO treatment. Robot-assisted laparoscopic pyeloplasty, treatment outcomes, and failed or complex cases are prominent recent research themes. Together, these findings provide an overview of how UPJO treatment research has developed and where recent activity has concentrated.

## Figures and Tables

**Table 1:** Top ten highly co-cited publications on the treatment of ureteropelvic junction obstruction.

Top Ten Highly Co-Cited Publications								
Rank	Author	Year	Journal	IF	JCR	Country	Co-Cited	Title
1	Inagaki T	2005	BJU International	4.5	Q1	United States	48	Laparoscopic pyeloplasty: current status [20]
2	Autorino R	2014	European Urology	25.2	Q1	United States	45	Robot-assisted and laparoscopic repair of ureteropelvic junction obstruction: a systematic review and meta-analysis [21]
3	Moon DA	2006	Urology	2.0	Q2	United Kingdom	35	Laparoscopic pyeloplasty: evolution of a new gold standard [22]
4	Lee RS	2006	Journal of Urology	7.5	Q1	United States	34	Pediatric robot assisted laparoscopic dismembered pyeloplasty: comparison with a cohort of open surgery [23]
5	Varda BK	2014	Journal of Urology	7.5	Q1	United States	26	National trends of perioperative outcomes and costs for open, laparoscopic and robotic pediatric pyeloplasty [24]
6	Braga LH	2009	European Urology	25.2	Q1	Canada	25	Systematic review and meta-analysis of robotic-assisted versus conventional laparoscopic pyeloplasty for patients with ureteropelvic junction obstruction: effect on operative time, length of hospital stay, postoperative complications, and success rate [25]
7	Metzelder ML	2006	Journal of Urology	7.5	Q1	Germany	25	Laparoscopic transabdominal pyeloplasty in children is feasible irrespective of age [26]
8	Bonnard A	2005	Journal of Urology	7.5	Q1	France	24	Retroperitoneal laparoscopic versus open pyeloplasty in children [27]
9	Jarrett TW	2002	Journal of Urology	7.5	Q1	United States	24	Laparoscopic pyeloplasty: the first 100 cases [28]
10	Schwentner C	2007	BJU International	4.5	Q1	Austria	24	Robotic Anderson-Hynes pyeloplasty: 5-year experience of one centre [29]

**Table 2:** Top ten publications by total citation count on the treatment of ureteropelvic junction obstruction.

Top Ten Publications by Total Citation Count								
Rank	Author	Year	Journal	IF	JCR	Country	Cited	Title
1	Xu N	2016	PLoS One	2.6	Q2	China	184	Comparison of retrograde balloon dilatation and laparoscopic pyeloplasty for treatment of ureteropelvic junction obstruction: results of a 2-year follow-up [30]
2	Autorino R	2014	European Urology	25.2	Q1	United States	184	Robot-assisted and laparoscopic repair of ureteropelvic junction obstruction: a systematic review and meta-analysis [21]
3	Chertin B	2006	European Urology	25.2	Q1	Israel	164	Conservative treatment of ureteropelvic junction obstruction in children with antenatal diagnosis of hydronephrosis: Lessons learned after 16 years of follow-up [31]
4	Moon DA	2006	Urology	2.0	Q2	United Kingdom	154	Laparoscopic pyeloplasty: Evolution of a new gold standard [22]
5	Link RE	2006	Annals of Surgery	6.5	Q1	United States	142	A prospective comparison of robotic and laparoscopic pyeloplasty [32]
6	Mufarrij PW	2008	Journal of Urology	7.5	Q1	United States	140	Robotic dismembered pyeloplasty: A 6-year, multi-institutional experience [33]
7	Braga LH	2009	European Urology	25.2	Q1	Canada	132	Systematic review and meta-analysis of robotic-assisted versus conventional laparoscopic pyeloplasty for patients with ureteropelvic junction obstruction: effect on operative time, length of hospital stay, postoperative complications, and success rate [25]
8	Yee DS	2006	Urology	2.0	Q2	United States	126	Initial comparison of robotic-assisted laparoscopic versus open pyeloplasty in children [34]
9	Mei H	2011	Journal of Endourology	2.8	Q2	China	109	Laparoscopic versus open pyeloplasty for ureteropelvic junction obstruction in children: A systematic review and meta-analysis [35]
10	Varda BK	2014	Journal of Urology	7.5	Q1	United States	103	National trends of perioperative outcomes and costs for open, laparoscopic and robotic pediatric pyeloplasty [24]

IF: 2024 Journal Impact Factor; JCR: 2024 Journal Citation Reports quartile.

**Table 3:** Literature-derived clinical context for treatment modalities in ureteropelvic junction obstruction (UPJO).

Treatment Modality	Core Description	Literature-Derived Advantages/Context	Limitations/Context	Examples of Populations Discussed in Cited Literature	Bibliometric/Clinical Context
Conservative observation	Serial clinical and imaging follow-up without immediate surgery.	Avoids unnecessary intervention in selected patients whose hydronephrosis and renal function remain stable.	Requires reliable follow-up; deterioration in drainage or renal function may prompt intervention.	Selected infants with mild prenatally detected hydronephrosis and preserved function [56].	Conservative management, including surveillance, remains represented in the clinical literature.
Open pyeloplasty	Open resection of the obstructed segment with reconstruction.	Long-established reconstructive approach with high reported success in clinical series and reviews [57,58].	Greater operative access and incision burden than minimally invasive approaches.	Used across age groups, including settings with complex anatomy or limited minimally invasive resources.	Foundational technique in the literature; bibliometric prominence has shifted toward minimally invasive approaches.
Laparoscopic pyeloplasty	Minimally invasive reconstruction performed through laparoscopic ports [59].	Clinical studies report reduced incision burden and favorable recovery compared with open surgery in selected patients.	Technically demanding suturing and a substantial learning curve.	Adults and older children in centers with advanced laparoscopic expertise.	An important transitional and continuing theme in minimally invasive UPJO research.
Robot-assisted pyeloplasty	Reconstructive pyeloplasty performed with a robotic platform.	Clinical literature describes three-dimensional visualization and wristed instrumentation and reports high success in selected cohorts.	Higher equipment cost and limited availability; working space can be constrained in small infants.	Adults and children, including selected complex or redo cases [60].	A prominent recent bibliometric theme; clinical studies assess perioperative and functional outcomes.
Endoscopic treatment (endopyelotomy/balloon dilation)	Incision or dilation of the obstructed segment through endoscopic or percutaneous access.	Less invasive access and relatively rapid recovery in selected patients.	Clinical reviews report lower and more variable success than reconstructive surgery, with recurrence dependent on anatomy and selection [61].	Selected short-segment or secondary obstruction and patients for whom reconstruction is less suitable.	Represents a distinct minimally invasive research cluster; not presented here as a first-line recommendation.
Reconstructive flap or substitution techniques	Use of local or adjacent tissue to bridge complex or long defects.	Can preserve the renal unit in selected complex reconstructions.	Technically demanding and supported mainly by specialized series.	Selected recurrent or complex UPJO with substantial ureteral defects [62].	Relevant to the recent literature on failed pyeloplasty and complex-case management.
Nephrectomy	Removal of a severely compromised renal unit.	Can be considered in selected patients with a poorly functioning, symptomatic renal unit after comprehensive assessment.	Irreversible loss of renal tissue.	Selected poorly functioning kidneys with clinical indications; thresholds vary and require individualized assessment [63].	A salvage option discussed in clinical literature, not a recommendation derived from bibliometric data.

## Data Availability

The harmonized bibliographic dataset and analysis parameter summary are available from the corresponding author upon reasonable request; redistribution of database-derived records may be limited by the licensing terms of the source databases.

## Supplementary Materials

The following supporting information can be downloaded at: https://www.mdpi.com/article/10.3390/healthcare14203533/s1. Table S1: Database-specific search strategies and search results; Table S2: Authors ranked by total citation count; Table S3: Institutions ranked by publication count; Table S4: Funding agencies by publication count; Table S5: The ten most productive journals with 2024 Journal Impact Factors and Journal Citation Reports quartiles; Table S6: Database-integration rules and network analysis settings.
