# Supplementary material for: Global Research Trends in Treatments for Ureteropelvic Junction Obstruction: A Multi-Database Bibliometric Analysis of the Past Two Decades
Source: Healthcare (Basel). 2026 Oct 20;14(20):3533. doi: 10.3390/healthcare14203533 (PMC13704246; doi:10.3390/healthcare14203533)
Supplement: Supplementary file 1 [file healthcare-14-03533-s001.zip › healthcare-4532550-supplementary.pdf]

Table S1 Detailed search strategies and outcomes.

Database	Query	Results
PubMed	#1: “ureteropelvic junction obstruction”[Title/Abstract] OR “ureteropelvic junction stenosis”[Title/Abstract] OR “pelviureteric junction obstruction”[Title/Abstract] OR “pelviureteric junction stenosis”[Title/Abstract] OR “ureteropelvic junction stricture”[Title/Abstract] OR “UPJO”[Title/Abstract] OR “PUJO”[Title/Abstract]	3,058
	“treatment”[Title/Abstract] OR “management”[Title/Abstract] OR “therapy”[Title/Abstract] OR “therapies”[Title/Abstract] OR “therapeutics”[Title/Abstract] OR “intervention”[Title/Abstract] OR “interventions”[Title/Abstract] OR “pyeloplasty”[Title/Abstract] OR “reconstruction”[Title/Abstract] OR “surgery”[Title/Abstract] OR “stent”[Title/Abstract] OR “laser”[Title/Abstract]	11,000,927
	#3: #1 AND #2	**2,414**
Embase	#1: ‘ureteropelvic junction obstruction’:ab,ti OR ‘ureteropelvic junction stenosis’:ab,ti OR ‘pelviureteric junction obstruction’:ab,ti OR ‘pelviureteric junction stenosis’:ab,ti OR ‘ureteropelvic junction stricture’:ab,ti OR ‘UPJO’:ab,ti OR ‘PUJO’:ab,ti	4,426
	#2: ‘treatment’:ab,ti OR ‘management’:ab,ti OR ‘therapy’:ab,ti OR ‘therapies’:ab,ti OR ‘therapeutics’:ab,ti OR ‘intervention’:ab,ti OR ‘interventions’:ab,ti OR ‘pyeloplasty’:ab,ti OR ‘reconstruction’:ab,ti OR ‘surgery’:ab,ti OR ‘stent’:ab,ti OR ‘laser’:ab,ti	15,235,452
	#3: #1 AND #2	**3,624**
Cochrane	#1: (ureteropelvic junction obstruction):ab,ti,kw OR (ureteropelvic junction stenosis):ab,ti,kw OR (pelviureteric junction obstruction):ab,ti,kw OR (pelviureteric junction stenosis):ab,ti,kw OR (ureteropelvic junction stricture):ab,ti,kw OR (UPJO):ab,ti,kw OR (PUJO):ab,ti,kw	141
	#2: (treatment):ab,ti,kw OR (management):ab,ti,kw OR (therapy):ab,ti,kw OR (therapies):ab,ti,kw OR (therapeutics):ab,ti,kw OR (intervention):ab,ti,kw OR (interventions):ab,ti,kw OR (pyeloplasty):ab,ti,kw OR (reconstruction):ab,ti,kw OR (surgery):ab,ti,kw OR (stent):ab,ti,kw OR (laser):ab,ti,kw	1,783,424
	#3: #1 AND #2	**137**
Scopus	#1: TITLE-ABS-KEY (“ureteropelvic junction obstruction” OR “ureteropelvic junction stenosis” OR “pelviureteric junction obstruction” OR “pelviureteric junction stenosis” OR “ureteropelvic junction stricture” OR “UPJO” OR	4,964

	"PUJO")	
	#2: TITLE-ABS-KEY ("treatment" OR "management" OR "therapy" OR "therapies" OR "therapeutics" OR "intervention" OR "interventions" OR "pyeloplasty" OR "reconstruction" OR "surgery" OR "stent" OR "laser")	20,963,599
	#3: #1 AND #2	**4,001**
Web of science	#1: TS = (ureteropelvic junction obstruction OR ureteropelvic junction stenosis OR pelviureteric junction obstruction OR pelviureteric junction stenosis OR ureteropelvic junction stricture OR UPJO OR PUJO)	3,763
	#2: TS = (treatment OR management OR therapy OR therapies OR therapeutics OR intervention OR interventions OR pyeloplasty OR reconstruction OR surgery OR stent OR laser)	13,244,422
	#3: #1 AND #2	**2,855**

All five databases were searched on January 20, 2026. The search strings were executed without language or document-type restrictions. The publication window (January 1, 2006, through December 31, 2025) was applied after export using the standardized publication-year field, while English-language and article/review eligibility criteria were applied during screening. Database-specific export formats were PubMed NBIB, Embase RIS, Scopus CSV, Web of Science plain text (Full Record and Cited References), and Cochrane RIS. Citation counts in Tables S2, S3, and S5 are based on the 736 publications matched to Web of Science.

Table S2 Top ten authors by citation count in UPJO treatment research.

Rank	Authors	Citation count	Publication count
1	Lorenzo, AJ	249	12
2	Noh, PH	207	9
3	Peters, CA	185	8
4	Gundeti, MS	165	12
5	Salle, JLP	165	7
6	Farhat, WA	163	6
7	El-Ghoneimi, A	157	6
8	Eden, CG	151	3
9	Casale, P	148	6
10	Braga, LHP	142	5

UPJO: ureteropelvic junction obstruction.

Table S3 Top ten institutions publishing research on the treatment of UPJO.

Rank	Institutions	Publication count	Citation count	Countries
1	Wroclaw Medical University	23	117	Poland
2	Peking University	21	69	China
3	University of Toronto	17	212	Canada
4	McMaster University	16	157	Canada
5	University of Michigan	16	153	United States
6	Capital Medical University	16	66	China
7	All India Institute of Medical Sciences	15	155	India
8	Huazhong University of Science and Technology	14	270	China
9	Hôpital Necker–Enfants Malades	14	87	France
10	Mansoura University	14	71	Egypt

UPJO: ureteropelvic junction obstruction.

Table S4 Top ten funding agencies supporting UPJO treatment research.

Rank	Funding Agencies	Publication count	Countries	Proportion
1	National natural science foundation of China	19	China	2.24%
2	United States department of health and human services	18	United States	2.12%
3	National institutes of health	16	United States	1.88%
4	Instituto politecnico nacional	7	Mexico	0.82%
5	NIH national institute of diabetes and digestive and kidney diseases	5	United States	0.59%
6	Grants-in-aid for scientific research	5	Japan	0.59%
7	Japan society for the promotion of science	4	Japan	0.47%
8	Ministry of education, culture, sports, science and technology	4	Japan	0.47%
9	National natural science foundation of Guangdong province	4	China	0.47%
10	National public welfare industry research projects	4	China	0.47%

UPJO: ureteropelvic junction obstruction. Percentages are calculated from the 849 included publications.

Table S5 Top ten journals publishing research on UPJO treatment.

Rank	Journal	IF	JCR	Publication count	Citation count
1	Journal of endourology	2.8	Q2	99	817
2	Journal of pediatric urology	1.9	Q2	87	779
3	Urology	2.0	Q2	76	630
4	Journal of urology	7.5	Q1	63	1345
5	Journal of laparoendoscopic & advanced surgical techniques	1.1	Q3	49	233
6	Frontiers in pediatrics	2.0	Q2	38	173
7	World journal of urology	2.9	Q2	33	208
8	Pediatric surgery international	1.6	Q2	27	157
9	International urology and nephrology	1.9	Q3	22	42
10	Urologia internationalis	1.3	Q3	19	98

Note: IF and JCR quartiles are from the 2024 Journal Citation Reports. UPJO: ureteropelvic junction obstruction.

Table S6 Reproducibility checklist for multi-database integration and bibliometric analysis.

Item	Required specification	Value used in this study
Search date	Exact calendar date on which all databases were searched	January 20, 2026
Export formats	Format exported from PubMed, Embase, Scopus, Web of Science, and Cochrane	PubMed: NBIB; Embase: RIS; Scopus: CSV; Web of Science: plain text (Full Record and Cited References); Cochrane: RIS
Initial record total	Total before deduplication; must equal sum of database-specific yields or be explicitly explained	13,031 (PubMed 2,414; Embase 3,624; Cochrane 137; Scopus 4,001; Web of Science 2,855)
EndNote automatic deduplication	Exact EndNote X9 comparison fields and sequence	EndNote X9 Find Duplicates configured on Author, Year, and Title. DOI, journal, and minor title/author variants were checked during manual verification.
Duplicate counts	Numbers removed automatically and manually	Total duplicates removed: 6,622. First-pass EndNote X9 duplicate detection removed 6,487 records; manual near-duplicate verification removed a further 135 records.
Near-duplicate handling	Procedure for missing DOI/minor title variants	Records without DOI or with minor title/author variants were manually compared using title, author, year, journal, and DOI when available.
Screening process	Title/abstract and full-text stages; reviewer agreement	After duplicate/document-type/year exclusions, two reviewers screened 2,640 titles/abstracts; 1,500 were excluded. Full text was assessed for 1,140 reports; 291 were

		excluded (224 not primarily treatment/management focused; 67 non-English). Formal kappa was not calculated; disagreements were resolved by consensus with a third reviewer.
Author-name harmonization	Rules for initials, hyphens, name variants, and homonyms	Surname plus initials; punctuation, spacing, and hyphen variants normalized; potential homonyms checked against affiliation and DOI when available.
Institution harmonization	Rules for department/hospital/university variants and mergers	Department/hospital variants mapped to a controlled parent-institution name when unambiguous; unresolved variants retained separately.
Journal-name harmonization	Full title vs abbreviation and title changes	Standardized to journal full title using ISSN/title metadata; abbreviation and punctuation variants merged.
Country harmonization	Country/territory naming rules	Standardized country labels (e.g., People's R China→China; England/Scotland/Wales→United Kingdom; Türkiye/Turkey→Turkey).
Missing fields	Rule for missing affiliation, country, funding, keywords, or cited references	Missing affiliation, funding, keyword, and cited-reference fields were not imputed; country was derived only from explicit affiliation information.
Citation-count source	Single database/source and date of citation extraction	Web of Science Core Collection; citation snapshot corresponding to the January 20,

		2026 analysis dataset.
Conflicting citation values	Rule used when duplicate records contained different citation counts	Citation values from other databases were not merged, summed, or averaged; Web of Science values were used for citation-based analyses.
Cited-reference source	Database/source used for cited-reference/co-citation fields	Web of Science Core Collection cited-reference field; records without Web of Science cited-reference data did not contribute to co-citation analyses.
Web of Science record linkage	Matching rule for retained publications	DOI was used first. Records without an exact DOI match were matched by title and checked against publication year and author information. Unresolved records remained unmatched, and citation data from other databases were not substituted.
Citation-analysis denominator	Number of included publications with Web of Science citation data	736 of 849 publications (86.7%); unmatched records remained in analyses that did not require citation data.
Co-citation denominator	Number of source publications with usable Web of Science cited-reference data	728 publications with a non-empty Web of Science Cited References field.
CiteSpace time slicing	Start/end year and years per slice	2006-2025; slice length = 1 year
CiteSpace term sources	Title, abstract, author keywords, Keywords Plus, etc.	Title; Abstract; Author Keywords; Keywords Plus/database-supplied keywords
CiteSpace node types	Node type used for each network	Country for country network; Institution for institution network; Author for author network; Reference for co-citation; Keyword

		for keyword co-occurrence/clustering/burst analyses
CiteSpace node selection	g-index (k) or Top N/Top N% criterion	g-index, k=25; LRF=3.0; L/N=10; LBY=5; e=1.0
CiteSpace pruning	Pathfinder, pruning sliced networks/merged network, or none	None for country, institution, author, and co-citation networks; Pathfinder for keyword network
Clustering	Clustering algorithm and label method (e.g., LLR, TF-IDF, MI)	CiteSpace clustering with log-likelihood ratio (LLR) labels. Co-citation timeline: Q=0.6578, S=0.8736. Keyword clustering: Q=0.7349, S=0.906.
Burst detection	Burst algorithm parameters/minimum duration	Kleinberg burst detection in CiteSpace; γ=1.0; minimum duration=2 years
bibliometrix v4.6	Functions and principal arguments used for descriptive/geographic analyses	convert2df(), biblioAnalysis(), summary(), and metaTagExtraction() for descriptive summaries/metadata. Annual trend figure: ggplot2/tidyverse. World map: sf, rnaturalearth, countrycode, scico, and ggplot2.
Journal metrics	JCR edition used for impact factor and quartile	2024 Journal Citation Reports
2026 references	Role of references published after analytic cutoff	Context only; not included in the 2006-2025 bibliometric dataset
Country network diagnostics	Network-specific values shown in CiteSpace output	N=55, E=221, density=0.1488; largest connected component=47 (85.5%); nodes labeled=1.0%; pruning=None.

Institution network diagnostics	Network-specific values shown in CiteSpace output	N=372, E=776, density=0.0112; largest connected component=188 (50.5%); nodes labeled=1.0%; pruning=None.
Author network diagnostics	Network-specific values shown in CiteSpace output	N=611, E=932, density=0.0050; largest connected component=23 (3.8%); nodes labeled=1.0%; pruning=None.
Co-citation network diagnostics	Network-specific values shown in CiteSpace output	N=787, E=4,002, density=0.0129; largest connected component=744 (94.5%); nodes labeled=1.0%; pruning=None; timeline Q=0.6578, S=0.8736.
Keyword network diagnostics	Network-specific values shown in CiteSpace output	N=518, E=1,037, density=0.0077; largest connected component=496 (95.8%); nodes labeled=1.0%; pruning=Pathfinder; clustering Q=0.7349, S=0.906.
